# Acupuncture for Treating Attention Deficit Hyperactivity Disorder in Children: A Systematic Review and Meta-Analysis

**DOI:** 10.3390/medicina59020392

**Published:** 2023-02-17

**Authors:** Lin Ang, Jung Tae Kim, Kibong Kim, Hye Won Lee, Jun-Yong Choi, Eunseop Kim, Myeong Soo Lee

**Affiliations:** 1 KM Science Research Division, Korea Institute of Oriental Medicine, Daejeon 34054, Republic of Korea; 2 Department of Korean Pediatrics, School of Korean Medicine, Pusan National University, Yangsan 50612, Republic of Korea; 3 I-MOM Korean Medicine Clinic, Jeju-si 63232, Republic of Korea; 4 Department of Korean Pediatrics, Korean Medicine Hospital, Pusan National University, Yangsan 50612, Republic of Korea; 5 KM Convergence Research Division, Korea Institute of Oriental Medicine, Daejeon 34054, Republic of Korea; 6 Department of Korean Internal Medicine, School of Korean Medicine, Pusan National University, Yangsan 50612, Republic of Korea; 7 Department of Korean Internal Medicine, Korean Medicine Hospital, Pusan National University, Yangsan 50612, Republic of Korea; 8 You and Green Korean Medicine Clinic, Daejeon 34113, Republic of Korea; 9 Korean Convergence Medical Science, University of Science and Technology, Daejeon 34113, Republic of Korea

**Keywords:** ADHD, acupuncture, complementary medicine, integrative medicine, systematic review

## Abstract

*Background and Objectives*: Attention-deficit hyperactivity disorder (ADHD) is a common childhood disorder characterized by inattention, hyperactivity, and impulsivity. However, it is uncertain whether the use of acupuncture (AT) in children with ADHD is supported by the current evidence. This review aims to provide updated evidence of the effectiveness of acupuncture in children with ADHD. *Methods*: Nine databases were searched from their inception to 28 July 2022. Two authors independently screened potentially eligible studies. The quality assessment of the selected studies was performed using Version 2 of the Cochrane risk-of-bias tool for randomized trials (RoB 2). The characteristics of the included studies were presented in a tabular form, and a meta-analysis was performed on the treatment effects of AT on ADHD symptoms. *Results*: Fourteen studies involving 1185 patients evaluating the efficacy of AT for ADHD treatment were included in this review. Compared to conventional medicine alone, the meta-analysis indicated that AT as an add-on to conventional medicine has a positive effect on improving conduct problems, learning problems, hyperactivity–impulsivity, and hyperactivity symptoms in ADHD patients. Similarly, AT alone was found to improve learning problems, hyperactivity–impulsivity, and hyperactivity symptoms in ADHD patients and exhibited better total treatment efficacy than conventional medicine alone. No major adverse events were reported. The risk of bias of the included studies was generally concerning. *Conclusions*: Evidence on the effectiveness of AT for ADHD patients is currently too limited to provide recommendations for its usage. More studies with the proper methodology are needed for the validation of AT interventions in treating children with ADHD.

## 1. Introduction

Attention-deficit hyperactivity disorder (ADHD) is a condition marked by an ongoing pattern of inattention, hyperactivity, and/or impulsive behaviors, such as extreme restlessness, trouble maintaining focus, and acting on impulse without self-control [1]. As one of the most common neurodevelopmental disorders of childhood, ADHD can interfere with the functioning or development of the child and can last into adulthood [2]. Although there is no known cause of ADHD and no known cure, there are therapies that may lessen symptoms and improve functioning, including medication, psychotherapy, training, education, or a combination of these treatments [3]. The most effective way to treat ADHD, in most cases, is by combining medication and behavior therapy [4].

Acupuncture is relatively easy, affordable, and safe compared to other conventional procedures used to treat ADHD. Many studies have shown that acupuncture has been used extensively to reduce primary symptoms in ADHD patients. Children with ADHD who receive acupuncture along with psychotherapy were reported to have improvements in their attention and reaction inhibition [5]. Acupuncture combined with methylphenidate drugs also showed symptom improvement in adults with ADHD [6]. The mechanism for the effect of acupuncture, however, is still being studied, as different manipulation techniques produce different needling sensations and therapeutic outcomes [7].

Several reviews have also been conducted on the use of acupuncture to treat ADHD in children and adolescents, of which two reviews [8,9] reported beneficial effects of acupuncture, and a Cochrane review [10] was unsuccessful in identifying any potential studies meeting the inclusion criteria. Nevertheless, these systematic reviews concluded that currently available data on the clinical effectiveness of acupuncture for treating ADHD are yet to be sufficient to support its routine use. This review aimed to provide updated evidence on the effectiveness of acupuncture in children with ADHD.

## 2. Methods

### 2.1. Study Registration

This review was registered at the Research Registry (unique identification number: reviewregistry1345), and its protocol was published. This review was reported in accordance with the PRISMA—Preferred Reporting Items for Systematic Reviews and Meta-analysis—guidelines [11].

### 2.2. Study Selection

#### 2.2.1. Types of Studies

We included only prospective randomized controlled trials (RCTs). Studies such as case-control, observational, cohort, qualitative, uncontrolled, laboratory, and case series studies were excluded. No restrictions were placed on the language or publication type.

#### 2.2.2. Types of Participants

Children who were below the age of 18 years [12] with ADHD were eligible for inclusion, regardless of sex and nationality.

#### 2.2.3. Types of Interventions and Controls

We included only invasive acupuncture interventions with or without electrical stimulation. Acupuncture interventions that only dealt with stimulating acupuncture points, such as acupressure, pressure buttons, and laser stimulation, were excluded. As comparators, we included interventions such as conventional medicine, sham acupuncture, and the waitlist.

#### 2.2.4. Types of Outcome Measures

Improvements in ADHD symptoms were selected as a primary outcome. As secondary outcomes, total treatment efficacy, quality of life, and adverse events (AEs) were selected. Outcomes had to be measured through tools that had psychometric properties.

### 2.3. Data Sources and Searches

#### 2.3.1. Databases and Other Searches

We searched the following databases from their inception until 28 July 2022: four English databases—PubMed, Embase, the Cochrane Central Register of Controlled Trials (CENTRAL), and the Allied and Complementary Medicine Database (AMED); one Chinese database—China National Knowledge Infrastructure (CNKI); and three Korean databases —KoreaMed, the Research Information Service System (RISS), and the Korean Studies Information Service System (KISS). We additionally searched the World Health Organization’s International Clinical Trial Registration Platform (ICTRP, https://www.who.int/clinical-trials-registry-platform accessed on 28 July 2022), ClinicalTrials.gov (https://clinicaltrials.gov/ accessed on 28 July 2022), and other relevant trial registries. We also checked all potential cross-references and reference lists of relevant reviews.

#### 2.3.2. Search Strategy

The following search terms were used: (acupuncture OR acup* OR electroacupuncture OR ear acupuncture) AND (attention deficit OR attention OR hyperactivity*). The search terms were adapted and/or translated for each database search.

### 2.4. Data Collection, Extraction, and Quality Assessment

#### 2.4.1. Data Extraction

The titles and abstracts were independently screened and assessed against the inclusion criteria by two authors (LA and KK). Any disagreements were referred for arbitration to a third author (MSL). Data were then extracted from the selected studies by two independent authors (LA and JTK). Data extracted included author(s) last name(s), year of publication, country, trial registration, sample size, age, sex, type of acupuncture intervention, type of control intervention, intervention regimen, acupuncture points used, outcome measures, ADHD diagnostic criteria, and AEs. We contacted the authors of the main trials by email when the reported data were insufficient.

#### 2.4.2. Quality Assessment

The quality of the included studies was assessed using Version 2 of the Cochrane risk-of-bias tool for randomized trials (RoB 2) [13]. Five bias domains were assessed: randomization process, deviations from intended interventions, missing outcome data, outcome measurements, and selection of reported results. Each domain was graded as low risk, some concerns, or high risk. Disagreements were resolved through discussion with a third author.

### 2.5. Data Analysis

Data analyses were performed using Review Manager (RevMan, Computer program, Version 5.4, The Cochrane Collaboration, 2020). The treatment effects are presented as mean differences (MDs) with 95% confidence intervals (CIs) for continuous outcomes and risk ratios (RRs) or odds ratios (ORs) with 95% CIs for dichotomous outcomes. Standardized mean differences (SMDs) with 95% CIs are presented for outcomes measured with different scales. Random-effects models were used in the meta-analysis due to the clinical heterogeneity of acupuncture interventions. The heterogeneity levels of the included studies were assessed using the chi-square test and Higgins I^2^ statistics. Subgroup analyses were not performed due to an insufficient number of studies included.

## 3. Results

### 3.1. Study Selection

**Figure 1:**
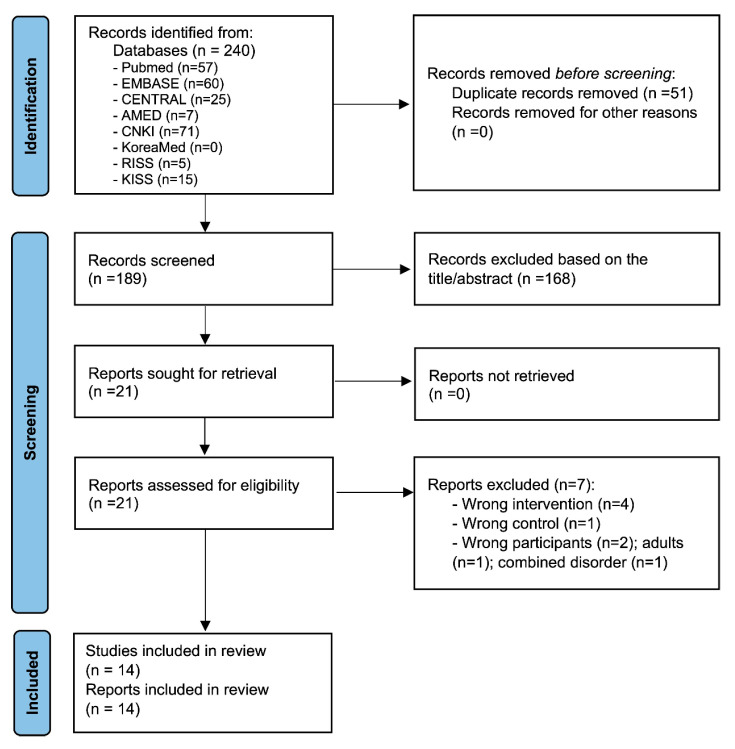
Study selection flow diagram.

Of the 240 articles screened, we reviewed the full texts of 21 studies. Fourteen studies met all inclusion criteria and were included for further analysis [14,15,16,17,18,19,20,21,22,23,24,25,26,27]. Seven studies were excluded for various reasons, of which four studies used interventions that did not meet our inclusion criteria (auricular acupuncture, behavior training, sensory integration therapy, etc.), one study used sham electroacupuncture (needle inserted in the same acupoints as the experimental group without an electric current passing through) as a control, one study included ADHD patients with tic disorders, and another study included adult patients. Figure 1 shows the study selection process based on predefined inclusion criteria.

### 3.2. Study Characteristics

Twelve of the fourteen studies were conducted in China [14,15,16,17,18,19,20,21,22,23,24,25], one was in Korea [27], and one was in Iran [26] (Table 1). Most of the studies used a parallel design, except for one study [27] that used a semi-crossover design. Except for two studies [26,27], most of the studies lacked trial registration details. The total sample size was 1185 children with ages ranging from 5.2 to 18.0 years, and 70% of the total study population was composed of boys. Eleven studies based their ADHD diagnosis on the *Diagnostic and Statistical Manual of Mental Disorders Fourth* or *Fifth Edition* (DSM-IV/V). None of the studies specified the subtype of ADHD. For acupuncture interventions, 13 studies used acupuncture with manual stimulation, and 1 study used electroacupuncture. The most commonly used acupuncture points were DU20 (Baihui), SP6 (Sanyinjiao), EM1 (Sishencong), EM2 (Yintang), and LV3 (Taichong), followed by DU24 (Shenting), HT7 (Shenmen), PC6 (Neiguan), KI3 (Taixi), and LI4 (Hegu). For control interventions, 12 studies used conventional medicine (methylphenidate hydrochloride oral or venlafaxine), 1 study used sham acupuncture (nonacupoints were used), and 1 study used a waitlist control.

### 3.3. Risk-of-Bias Assessment

**Figure 2:**
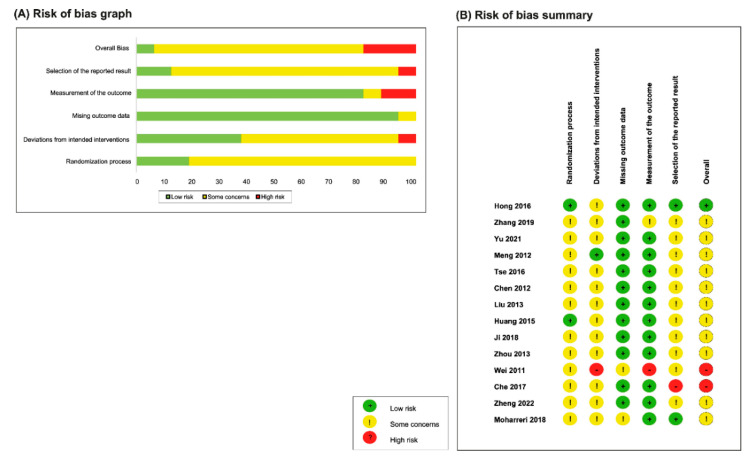
Risk of bias. (**A**) Risk-of-bias graph. (**B**) Risk-of-bias summary [14,15,16,17,18,19,20,21,22,23,24,25,26,27].

The risk-of-bias graph and summary for the included studies are outlined in Figure 2. For the randomization process, only two studies [20,27] were evaluated as having a low risk of bias, as the study participants were both adequately randomized and concealed. The remaining studies were rated as concerning; six studies [9,10,13,17,19,20] only adequately randomized the study participants but did not provide information on allocation concealment, and six studies [8,11,12,15,16,18] only described the trial as randomized without additional information on the methods of randomization or allocation concealment.

For the bias due to deviations from the intended interventions, one study was judged as low risk [27], one study [23] was judged as high risk, and the remaining studies were rated as concerning. One trial [27] was an open-label trial, and the remaining studies did not provide any information on blinding. Most of the studies performed intention-to-treat analyses, except for two studies [25,26], and one study [23] did not provide sufficient information for judgment, as there were no numerical results reported. For missing outcome data, only two studies were judged as concerning, and the remaining studies were low-risk. One study [26] was reported to have a considerably high dropout rate.

Regarding the bias associated with the measure of outcomes, one study [23] was judged as high-risk due to improper reporting. Another study [18] was judged concerning, as the method of outcome measurement was not reported. The bias in the selection of the reported results was judged as low in two studies [26,27], as both outcome measures and analyses were consistent with their trial protocols, and high in one [24], as discrepancy reporting of the sample size was found. The remaining studies were judged as concerning, as trial protocols are not available. Overall, the risk of bias was judged as low in 1 study [27], high in 2 studies [23,24], and concerning in the remaining 11 studies.

### 3.4. Effects of Interventions

#### 3.4.1. Acupuncture vs. Conventional Medicine 

##### Inattention

One study [20] compared acupuncture alone with conventional medicine and reported that acupuncture showed a favorable effect on reducing the symptoms of inattention in children with ADHD (n = 68, MD −5.16, CI 95% −6.94 to −3.38, *p* < 0.00001) compared to the effects of conventional medicine. 

##### Hyperactivity–Impulsivity

**Figure 3:**
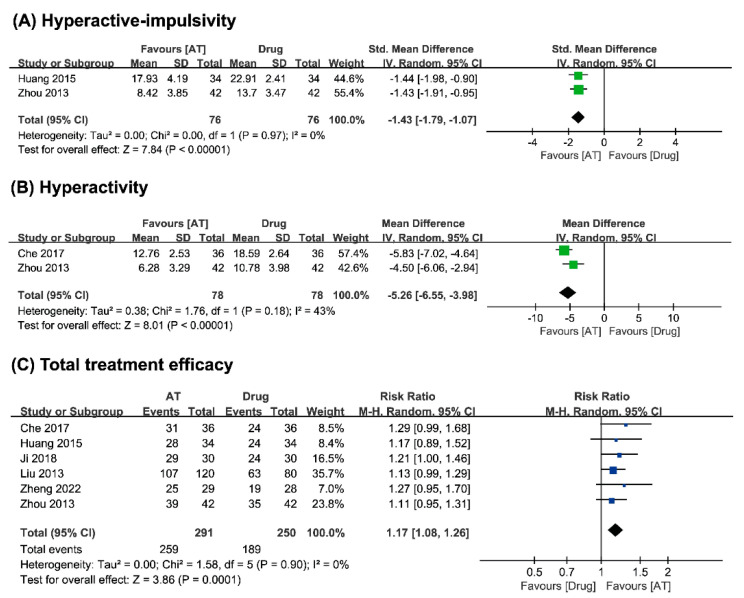
Forest plot of (**A**) hyperactivity–impulsivity, (**B**) hyperactivity, (**C**) total treatment efficacy of acupuncture vs. conventional medicine.

Two studies [20,22] compared acupuncture with conventional medicine and reported that acupuncture reduced the symptoms of hyperactivity–impulsivity. The meta-analysis showed that acupuncture has a positive effect on reducing hyperactivity–impulsivity in children with ADHD (n = 152, SMD −1.43, CI 95% −1.79 to −1.07, *p* < 0.00001, I^2^ = 0%, Figure 3A) compared to the effect of conventional medicine alone.

##### Hyperactivity

Two studies [22,24] comparing acupuncture alone with conventional medicine reported that acupuncture reduced the symptoms of hyperactivity in children with ADHD. The meta-analysis showed that acupuncture has a positive effect on reducing hyperactivity (n = 156, MD −5.26, CI 95% −6.55 to −3.98, *p* < 0.00001, I^2^ = 43%, Figure 3B) compared to the effect of conventional medicine.

##### Conduct Problems

One study [22] compared acupuncture with conventional medicine and showed a positive effect on reducing conduct problems in children with ADHD (n = 84, MD 8.25, CI 95% 4.76 to 11.74, *p* < 0.00001).

##### Learning Problems

One study [22] compared acupuncture with conventional medicine and showed a positive effect on reducing learning problems in children with ADHD (n = 84, MD 9.70, CI 95% −5.44 to 13.96, *p* < 0.00001).

##### Total Treatment Efficacy

Six studies [19,20,21,22,24,25] comparing acupuncture alone with conventional medicine reported that acupuncture has beneficial effects on children with ADHD. The meta-analysis showed that acupuncture has a positive effect on total treatment efficacy (n = 541, RR 1.17, 95% CI 1.08 to 1.26, *p* = 0.0001, I^2^ = 0%, Figure 3C) compared to the effects of conventional medicine.

#### 3.4.2. Acupuncture Plus Conventional Medicine vs. Conventional Medicine

##### Hyperactivity–Impulsivity

**Figure 4:**
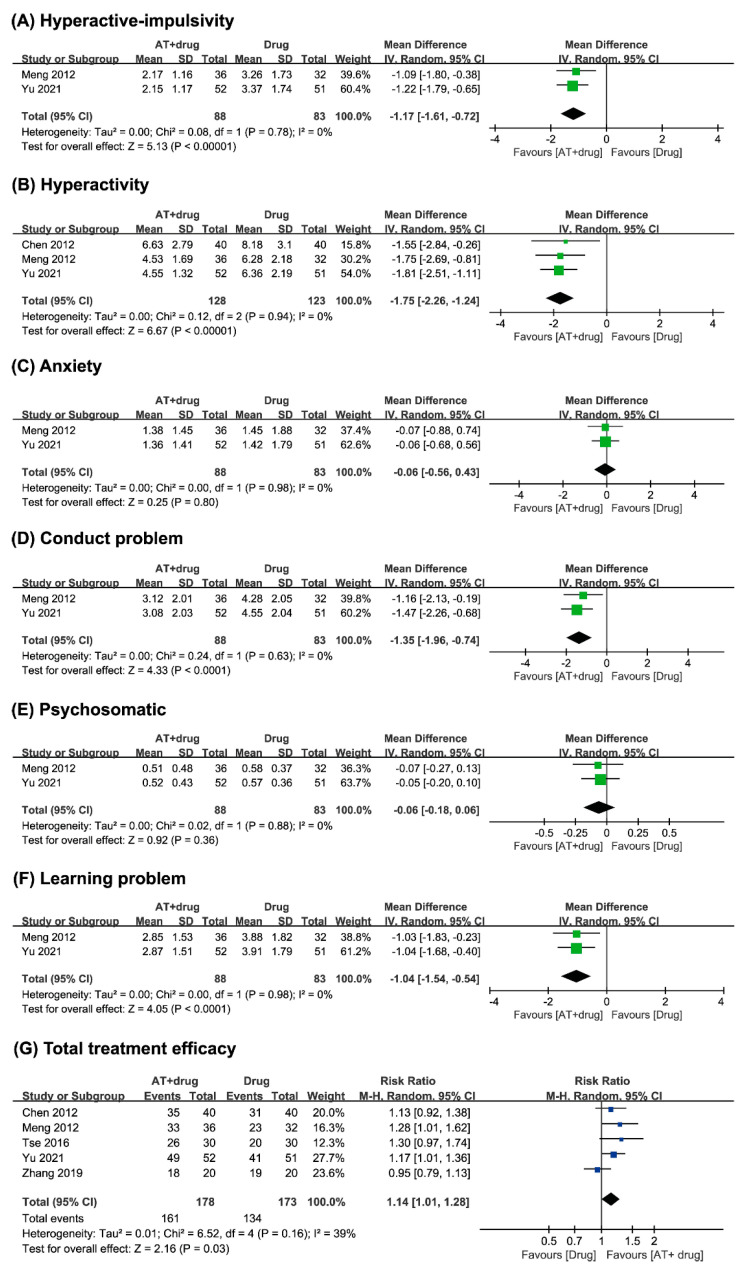
Forest plot of (**A**) hyperactivity–impulsivity, (**B**) hyperactivity, (**C**) anxiety, (**D**) conduct problems, (**E**) psychosomatic, (**F**) learning problems, and (**G**) total treatment efficacy of acupuncture plus conventional medicine vs. conventional medicine.

Two studies [14,17] comparing acupuncture complementing conventional medicine with conventional medicine alone reported that acupuncture as a complement reduced the symptoms of hyperactivity–impulsivity in children with ADHD. The meta-analysis showed that acupuncture complementing conventional medicine has a positive effect on reducing hyperactivity–impulsivity (n = 171, MD −1.17, CI 95% −1.61 to −0.72, *p* < 0.00001, I^2^ = 0%, Figure 4A) compared to conventional medicine alone.

##### Hyperactivity

Three studies [8,9,11] comparing acupuncture complementing conventional medicine with conventional medicine alone reported that acupuncture as a complement reduced the symptoms of hyperactivity in children with ADHD. The meta-analysis showed that acupuncture complementing conventional medicine has a positive effect on reducing hyperactivity (n = 251, MD −1.75, CI 95% −2.26 to −1.24, *p* < 0.00001, I^2^ = 0%, Figure 4B) compared to conventional medicine alone.

##### Anxiety

Two studies [14,17] comparing acupuncture complementing conventional medicine with conventional medicine alone reported that acupuncture as a complement did not significantly reduce the symptoms of anxiety in children with ADHD. The meta-analysis showed that acupuncture complementing conventional medicine has an equivalent effect on reducing anxiety (n = 171, MD −0.06, CI 95% −0.56 to 0.43, *p* = 0.80, I^2^ = 0%, Figure 4C) compared to the effect of conventional medicine alone.

##### Conduct Problems

Two studies [14,17] comparing acupuncture complementing conventional medicine with conventional medicine alone reported that acupuncture as a complement improved behavior in children with ADHD. The meta-analysis showed that acupuncture complementing conventional medicine has a positive effect on reducing conduct problems (n = 171, MD −1.35, CI 95% −1.96 to −0.74, *p* < 0.0001, I^2^ = 0%, Figure 4D) compared to the effect of conventional medicine alone.

##### Psychosomatic

Two studies [14,17] comparing acupuncture complementing conventional medicine with conventional medicine alone reported that acupuncture as a complement did not significantly improve mental health in children with ADHD. The meta-analysis showed that acupuncture complementing conventional medicine has an equivalent effect on reducing psychosomatic scores (n = 171, MD −0.06, CI 95% −0.18 to 0.06, *p* = 0.36, I^2^ = 0%, Figure 4E) compared to the effect of conventional medicine alone.

##### Learning Problems

Two studies [14,17] comparing acupuncture complementing conventional medicine with conventional medicine alone reported that acupuncture as a complement improved learning ability in children with ADHD. The meta-analysis showed that acupuncture complementing conventional medicine has a positive effect on reducing learning problems (n = 171, MD −1.04, CI 95% −1.54 to −0.54, *p* < 0.0001, I^2^ = 0%, Figure 4F) compared to the effect of conventional medicine.

##### Total Treatment Efficacy

Five studies compared acupuncture complementing conventional medicine with conventional medicine alone, where three studies [8,10,11] reported that acupuncture as a complement has beneficial effects on children with ADHD, and two studies [15,18] reported otherwise. The meta-analysis showed that acupuncture complementing conventional medicine has an equivalent effect on total treatment efficacy (n = 351, RR 1.14, 95% CI 1.01 to 1.28, *p* = 0.03, I^2^ = 39%, Figure 4G) compared to the effect of conventional medicine alone.

#### 3.4.3. Acupuncture Plus Conventional Medicine vs. Sham Acupuncture Plus Conventional Medicine

##### Inattention

One study [26] compared acupuncture with sham acupuncture and showed an equivalent effect on reducing hyperactivity–impulsivity in children with ADHD (n = 59, MD −1.95, CI 95% −4.90 to 1.00, *p* = 0.19).

##### Hyperactivity–Impulsivity

One study [26] compared acupuncture with sham acupuncture and showed an equivalent effect on reducing hyperactivity–impulsivity in children with ADHD (n = 59, MD 0.88, CI 95% −1.94 to 3.70, *p* = 0.54).

#### 3.4.4. Adverse Events (AEs)

Six studies assessed AEs in their trials; one study [26] reported that no AEs were found, five studies [9,12,13,19,21] reported minor AEs, and the remaining eight studies did not report AEs. The details of the AEs are listed in Table 2.

## 4. Discussion

### 4.1. Summary of Main Results

This systematic review included 14 studies of 1185 patients assessing the effectiveness of AT for ADHD treatment. Our findings show that AT complementing conventional medicine has favorable effects on improving conduct problems, learning problems, hyperactivity–impulsivity, and hyperactivity symptoms in ADHD patients compared to the effects of conventional medicine alone. Likewise, AT alone compared with conventional medicine alone improves conduct problems, learning problems, hyperactivity–impulsivity, and hyperactivity symptoms, as well as inattention and total treatment efficacy, in ADHD patients. However, AT complementing conventional medicine did not show beneficial effects on improving anxiety, psychosomatic scores, and total treatment efficacy compared with the effects of conventional medicine alone. On the other hand, AT complementing conventional medicine also showed equivalent effects on improving inattention and hyperactivity–impulsivity compared to the effects of sham AT with conventional medicine. The risk of bias of the included studies was generally concerning. Evidence on the effectiveness of AT for ADHD patients is currently too limited to provide recommendations for its usage.

### 4.2. Overall Completeness and Applicability of Evidence

In this review, there is a lack of consistency in the measurement of outcomes, which makes it difficult to pool the results on the effectiveness of AT. Several instruments were used across the studies to measure the symptoms of ADHD, including the Conners Parent Symptoms Questionnaire (PSQ), Attention-Deficit Hyperactivity Disorder Rating Scale (ADHD-RS), and the Swanson, Nolan, and Pelham Teacher and Parent Rating Scale (SNAP-IV). Although many studies used the Conners PSQ to rate ADHD symptoms, different subscales were reported across the studies, limiting further analysis of the results.

Additionally, the quality of the studies included in this systematic review was poor. Assessing the blinding of studies is a major aspect in determining the risk of bias of a study, but most of the studies did not provide any relevant information. Although it is understandable that blinding could be difficult to achieve in behavioral-type and invasive intervention studies, the authors should provide reasoning on how bias is being avoided in their study. Therefore, the positive findings in this review may be compromised and should be interpreted cautiously.

### 4.3. Agreements and Disagreements with Other Reviews

Two review articles assessing the effectiveness of AT as a treatment option for ADHD were published in 2011 [8,9]. One review included only three studies with different types of AT interventions and comparators [9], and one Cochrane review included none [10]. Both reviews concluded that there is limited evidence to support the use of AT for ADHD. A recent review included 10 studies with 876 ADHD patients but selected only the effective rate and hyperactivity scores as their main outcomes and suggested that AT may be more beneficial than methylphenidate hydrochloride [8]. In this review, a more comprehensive search was performed, and more studies were included. The effectiveness of AT alone or as an add-on to conventional medicine in treating ADHD was also evaluated according to multiple outcomes.

### 4.4. Limitations

This review focused on AT interventions implemented to improve symptoms in children with ADHD. First, there were insufficient data on several clinical outcomes, which limits the pooling of results; therefore, the statistical power of the meta-analysis in this review is fairly low. Second, the quality of the RCTs included was concerning, which may decrease the validity of positive findings. Many included studies did not describe randomization methods, blinding, or follow-ups. Third, the studies included in this review studied a wide range of ages, but it is difficult to perform subgroup analysis on children and adolescents due to the lack of studies. Although none of the studies included in this review reported serious acupuncture-related AEs, more than half of the included studies did not assess the incidence of AEs. Future studies on acupuncture should investigate the incidence of AEs and their causality with acupuncture treatment.

### 4.5. Implications for Practice and Research

The current evidence on AT is still too limited to support its routine use in treating ADHD. The methodology of the included studies needs to be greatly improved. A study with a larger sample size with adequate allocation concealment and blinding, appropriate outcome measurement instruments, and a safety evaluation should be performed to validate and provide more evidence. Future studies should also include longer follow-ups to enable the evaluation of the short- and long-term benefits of the AT intervention. Additionally, the findings of the studies should be properly reported according to the Consolidated Standards of Reporting Trials (CONSORT) statement and the Standards for Reporting Interventions in Controlled Trials of Acupuncture (STRICTA) guideline to ensure the transparency and quality of the reported trials.

## 5. Conclusions

This review shows that there is limited evidence to support the use of AT as a treatment for ADHD in children despite exhibiting several promising effects on symptom improvement. To integrate it into routine clinical practice, future studies should adopt the proper methodology to increase the quality of evidence.

## Figures and Tables

**Table 1:** Study characteristics of the included trials.

Study ID	Sample Size Gender (M/F)	Age Disease Course (I/C, yr.)	Intervention Group (Regimen, n)	Control Group (Regimen, n)	Acupuncture Points (Main Points)	Outcome Measures/ Rating Scale Used	Results (MD/RR [95%CI], *p*-Value)
Meng 2012 [14]	68 52/16	9/9 5.2/5.3	(A) AT (3 times per week for 12 weeks, n = 36), plus B	(B) CM (methylphenidate hydrochloride ER tablets, once a day, n = 32)	DU20, DU24, GB13(bil), EM1, PC6, HT7, SP6, KI3, LV3	(1) PSQ score (2) Treatment efficacy	(1) Anxiety: MD −0.07 [−0.88, 0.74], NS; conduct problems: MD −1.16 [−2.13, −0.19], *p* = 0.02; hyperactivity: MD −1.75 [−2.69, −0.81], *p* = 0.0002; hyperactivity–impulsivity: MD −1.09 [−1.80, −0.38], *p* = 0.003; learning problems: MD −1.09 [−1.80, −0.38], *p* = 0.003; psychosomatic: MD −0.07 [−0.27, 0.13], NS (2) RR 1.28 [1.01, 1.62], *p* = 0.05
Chen 2012 [15]	120 86/34	8.6/8.8 2.1/2.3	(A) AT (2–3 times a week for 12 weeks, n = 40), plus B	(B) CM (methylphenidate hydrochloride ER tablets, once a day, n = 40) *(C) Behavioral therapy, plus B (n = 40)*	EM2, DU23, DU20, PC6, LI4, DU26, ST36, KI3, SP6, LV3	(1) PSQ score (2) Treatment efficacy	(1) A vs. B: hyperactivity: MD −5.83 [−7.02, −4.64], *p* < 0.00001 (2) A vs. B: RR 1.13 [0.92, 1.38], NS
Tse 2016 [16]	60 35/25	8.4/9.5 2.5/2.0	(A) AT (5 times per week for 24 weeks, n = 30), plus B	(B) CM (methylphenidate hydrochloride ER tablets, twice a day, n = 30)	*Fang*’s scalp acupuncture: Bregma, Lambda, *Fuxiang tou, Fuzang shangjiao, Siwei, Jiyi, Yunping*	(1) PSQ score (2) SNAP-IV score (3) CRT score (4) Treatment efficacy	(1) Inappropriate reporting (2) MD −3.37 [−5.92, −0.82], *p* = 0.010 (3) MD 7.85 [5.43, 10.27], *p* < 0.00001 (4) RR 1.30 [0.97, 1.74], NS
Yu 2021 [17]	103 92/11	9.5/9.5 3.2/4.3	(A) AT (3 times per week for 4 weeks, n = 52), plus B	(B) CM (venlafaxine, once daily, n = 51)	DU20, DU24, GB13(bil), EM1, PC6, HT7, SP6, KI3, LV3	(1) PSQ score (2) Treatment efficacy	(1) Anxiety: MD −0.06 [−0.68, 0.56], NS; conduct problems: MD −1.47 [−2.26, −0.68], *p* = 0.0002; hyperactivity: MD −1.81 [−2.51, −1.11], *p* < 0.00001; hyperactivity–impulsivity: MD −1.22 [−1.79, −0.65], *p* < 0.0001; learning problems: MD −1.04 [−1.68, −0.40], *p* = 0.001; psychosomatic: MD −0.05 [−0.20, 0.10], NS (2) RR 1.17 [1.01, 1.36], *p* = 0.04
Zhang 2019 [18]	40 27/13	8.5 n.r.	(A) AT (1 or 2 times per week for 30 therapies, n = 20), plus B	(B) CM (methylphenidate hydrochloride ER tablets, once daily, n = 20)	EM1, DU24, GB13(bil), EM2, GB14 (bil)	(1) Treatment efficacy (2) Behavioral score * (3) Hyperactivity index *	(1) RR 0.95 [0.79,1.13], NS (2) MD 9.11 [7.19, 11.03], *p* < 0.00001 (3) MD 2.25 [0.81, 3.69], *p* = 0.002
Liu 2013 [19]	200 131/69	6–14 n.r.	AT (3 times per week for 12 weeks, n = 120)	CM (methylphenidate hydrochloride ER tablets, twice a day, n = 80)	PC6, DU26, SP6, Du20, EM2, DU23, HT7, PC7	(1) Treatment efficacy (2) Symptom disappearance rate	(1) RR 1.13 [0.99, 1.29], NS (2) Inappropriate reporting, NS
Huang 2015 [20]	68 35/33	6–13 0.5–<1	AT (3 times per week for 8 weeks, n = 34)	CM (methylphenidate hydrochloride ER tablets, once a day, n = 34)	DU14, DU1, CV15	(1) SNAP-IV score (2) CRT score (3) Treatment efficacy	(1) Total: MD −8.61 [−10.59, −6.63], *p* < 0.00001; hyperactivity–impulsivity: MD −4.98 [−6.60, −3.36], *p* < 0.00001; inattention: MD −5.16 [−6.94, −3.38], *p* < 0.00001; others: MD −2.25 [−4.38, −0.12], *p* = 0.04 (2) MD −0.70 [−4.72, 3.32], NS (3) RR 1.17 [0.89, 1.52], NS
Ji 2018 [21]	60 36/24	7.6/8.4 0.2–4	AT (10 times, n = 30)	CM (methylphenidate hydrochloride ER tablets, twice per day, n = 30)	DU20, DU17, EM1, DU24, EM2, ST36	Treatment efficacy	RR 1.21 [1.00, 1.46], *p* = 0.05
Zhou 2013 [22]	84 64/20	8.9/8.8 1.9/1.8	AT (3 times per week for 12 weeks, n = 42)	CM (methylphenidate hydrochloride ER tablets, twice a day, n = 42)	PC6, DU24, SP6, HT7, PC7	(1) PSQ score (2) Treatment efficacy	(1) Conduct problems: MD 8.25 [4.76, 11.74], *p* < 0.00001; hyperactivity: MD −4.50 [−6.06, −2.94], *p* < 0.00001; hyperactivity–impulsivity: MD −5.28 [−6.85, −3.71], *p* < 0.00001; learning problems: MD 9.70 [5.44, 13.96], *p* < 0.00001 (2) RR 1.11 [0.95, 1.31], NS
Wei 2011 [23]	70 38/32	10.8 n.r.	AT (3 times per week for 4 weeks, n = 35)	CM (methylphenidate hydrochloride ER tablets, twice a day, n = 35)	DU14, CV8	(1) Rating score * (2) Treatment efficacy	(1) Inappropriate reporting, *p* < 0.05 (2) Inappropriate reporting, NS
Che 2017 [24]	72 11/7 *	9.2/7.6 0.8–3.2	AT (once daily for 4 weeks, n = 36)	CM (Methylphenidate hydrochloride ER tablets, n.r., n = 36)	LV3, KI3, SP6, GB34, LI4, *Dacha*, HT7, GB-20, EM5, EM2, EM1	(1) PSQ score (2) Treatment efficacy	(1) Hyperactivity: MD −1.55 [−2.84, −0.26], *p* = 0.02 (2) RR 1.29 [0.99, 1.68], NS
Zheng 2022 [25]	57 40/17	11/11 3.0/3.1	EA (5 times per week for 12 weeks, n = 30)	Methylphenidate hydrochloride ER tablets, once a day (n = 30)	DU26, EM2, DU20, DU14, BL15 (bil), BL17 (bil), BL18 (bil), BL20 (bil), BL23 (bil), ST25 (bil), CV6, CV4, ST29 (bil)	Treatment efficacy	RR 1.27 [0.95, 1.70], NS
Moharreri 2018 [26]	90 57/7	10.4/10.9 n.r.	AT (3 times per week for 4 weeks, n = 51), plus CM ^✝^	Sham acupuncture (3 times per week for 4 weeks, n = 39), plus CM ^✝^	BL18, BL20, BL23, KI3, LV3, SP6, LI4, CV17, CV12, DU24, DU20, EM2	(1) ADHD-RS score (2) Visual CPT score	(1) Total: MD −1.06 [−6.28, 4.16], NS; hyperactivity–impulsivity: MD 0.88 [−1.94, 3.70], NS; inattention: MD −1.95 [−4.90, 1.00], NS (2) Correct hit: MD 3.24 [−3.05, 9.53], NS; omission error: MD −4.30 [−7.68, −0.92], *p* = 0.01; commission error: MD 1.06 [−3.65, 5.77], NS; reaction time: MD −24.06 [−88.96, 40.84], NS
Hong 2016 [27]	93 78/15	10.9/11.1 n.r.	AT (twice per week for 6 weeks, n = 46)	Waitlist (n = 47)	DU20, EM1, LI4 (bil), LI11 (bil), SP6 (bil), LV3 (bil)	(1) ADHD-RS score ^‡^ (2) PSQ score ^‡^ (3) IOWA-RS score ^‡^ (4) CGI-S score ^‡^ (5) FAIR score ^‡^ (6) CBCL score ^‡^ (7) CNT score ^‡^ (8) CPT ^‡^ (9) CCPT ^‡^	(1) Total: NS; Hyperactivity–impulsivity: NS; inattention: NS (2–3) NS (4) *p* = 0.012 (5) P: NS; Q: *p* = 0.022; C: NS (6) Total: NS; ADHD subscale: NS; external subscale: NS (7) Digit span test: forward: NS, backward: *p* = 0.027; visual span test: forward: NS, backward: *p* = 0.03; verbal learning test: *p* = 0.007 (8) Auditory—correct hit: NS; omission error: NS; commission error: NS; reaction time: *p* = 0.011; visual—correct hit: NS; omission error: NS; commission error: NS; reaction time: *p* = 0.004 (9) Auditory: Correct hit: NS, Omission error: NS, Commission error 1: NS, Commission error 2: NS, Reaction time: NS; Visual: Correct hit: NS, Omission error: NS, Commission error 1: NS, Commission error 2: NS, Reaction time: NS

ADHD-RS, Attention-Deficit Hyperactivity Disorder Rating Scale; AT, acupuncture; bil, bilateral; CBCL, Child Behavior Checklist; CGI-S, Clinical Global Impression-Severity rating scale; CM, conventional medication; CNT, Computerized Neurocognitive Function Test; CCPT, Controlled Continuous Performance Test; CPT, Continuous Performance Test; CRT, Combined Raven’s test; EA, electroacupuncture; ER, extended-release; FAIR, Frankfurt Attention Inventory; n.r., not reported; PSQ, Conners Parent Symptom Questionnaire; SNAP-IV, Swanson, Nolan, and Pelham Teacher and Parent Rating Scale; ^✝^, methylphenidate/methylphenidate and clonidine; *, scale used for outcome measurement not reported; ^‡^, only post–pre results presented.

**Table 2:** Details of the adverse events reported in the included studies.

Study ID	AEs
Meng 2012 [14]	n.r.
Chen 2012 [15]	Loss of appetite (AT, 2; CM, 7), headache (AT,1; CM, 1), insomnia (AT, 1; CM, 5), abdominal pain (CM, 2)
Tse 2016 [16]	n.r.
Yu 2021 [17]	n.r.
Zhang 2019 [18]	Vomiting, hallucinations, agitation, hyperreflexia, muscle twitches, convulsions, euphoria, tremor, confusion, delirium, sweating, flushing, headache, high fever, tachycardia, palpitations, hypertension, mydriasis, arrhythmia, dry mouth, etc. (AT/CM: n = 5/6) n.r. in details
Liu 2013 [19]	Nausea, dry mouth, constipation, loss of appetite, etc. (n = 15) Not differentiated according to intervention
Huang 2015 [20]	n.r.
Ji 2018 [21]	n.r.
Zhou 2013 [22]	n.r.
Wei 2011 [23]	n.r.
Che 2017 [24]	n.r.
Zheng 2022 [25]	Loss of appetite (CM, 6), insomnia (CM, 4), headache (CM, 3), abdominal pain (CM, 1), muscle twitches (CM, 1)
Moharreri 2018 [26]	None
Hong 2016 [27]	Mild headaches (AT, 3)

AT: acupuncture; CM: conventional medicine; n.r.: not reported.

## Data Availability

Not applicable.
